# Whole Genome Sequence of the Parasitoid Wasp *Microplitis demolitor* That Harbors an Endogenous Virus Mutualist

**DOI:** 10.1534/g3.118.200308

**Published:** 2018-07-17

**Authors:** Gaelen R. Burke, Kimberly K. O. Walden, James B. Whitfield, Hugh M. Robertson, Michael R. Strand

**Affiliations:** * Department of Entomology, University of Georgia, Athens, GA, 30602; † Department of Entomology, University of Illinois, Urbana-Champaign, Urbana, IL, 61801

**Keywords:** symbiosis, Hymenoptera, Braconidae, *Microplitis demolitor* bracovirus (MdBV), *Polydnaviridae*

## Abstract

*Microplitis demolitor* (Hymenoptera: Braconidae) is a parasitoid used as a biological control agent to control larval-stage Lepidoptera and serves as a model for studying the function and evolution of symbiotic viruses in the genus *Bracovirus*. Here we present the *M. demolitor* genome (assembly version 2.0), with a genome size of 241 Mb, and a N50 scaffold and contig size of 1.1 Mb and 14 Kb, respectively. Using RNA-Seq data and manual annotation of genes of viral origin, we produced a high-quality gene set that includes 18,586 eukaryotic and 171 virus-derived protein-coding genes. Bracoviruses are dsDNA viruses with unusual genome architecture, in which the viral genome is integrated into the wasp genome and is comprised of two distinct components: proviral segments that are amplified, circularized, and packaged into virions for export into the wasp’s host via oviposition; and replication genes. This genome assembly revealed that at least two scaffolds contain both nudivirus-like genes and proviral segments, demonstrating that at least some of these components are near each other in the genome on a single chromosome. The updated assembly and annotation are available in several publicly accessible databases; including the National Center for Biotechnology Information and the Ag Data Commons. In addition, all raw sequence data available for *M. demolitor* have been consolidated and are available for visualization at the i5k Workspace. This whole genome assembly and annotation represents the only genome-scale, annotated assembly from the lineage of parasitoid wasps that has associations with bracoviruses (the ‘microgastroid complex’), providing important baseline knowledge about the architecture of co-opted virus symbiont genomes.

*Microplitis demolitor* (Hymenoptera: Braconidae) is a parasitoid wasp species that completes its immature stages of development in larval Lepidoptera. It is endemic to Queensland, Australia, and was first introduced to the United States in 1983 for biological control of *Helicoverpa zea* and select other lepidopteran pests (Shepard *et al.* 1983; Burke 2016). It has become an important system for studying the function and evolution of mutualistic symbiotic viruses in the genus *Bracovirus* (family *Polydnaviridae*). Bracoviruses are produced in wasp ovaries in the nuclei of specialized (calyx) cells, which lyse and release virions into the reproductive tract where they form a paste-like “calyx fluid” (Stoltz and Vinson 1979). During parasitism, the female wasp uses her ovipositor to inject eggs and calyx fluid into the body of the host insect, where the virions immediately infect host cells, particularly host blood or hemocyte cells (Beck *et al.* 2007; Strand *et al.* 1992; Strand 1994). The virions carry circular double-stranded DNA molecules that are injected into host cell nuclei where virulence genes are transcribed rapidly after parasitism (Stoltz and Vinson 1979; Strand *et al.* 1992). The protein products of virulence genes are involved in suppressing the host immune system and altering host development to favor the survival of the wasp egg and larva (Strand and Burke 2014).

Bracoviruses (BVs) evolved approximately 100 million years ago from an ancestral virus in the family *Nudiviridae* (Bézier *et al.* 2009; Murphy *et al.* 2008). Today, all known BVs persist in wasps as integrated proviruses. BV genome architecture is unusual because genes are dispersed in the wasp genome and organized in ways that enable formation of replication-defective virions that wasps use to infect hosts (Bézier *et al.* 2009; Bézier *et al.* 2013; Burke *et al.* 2014; Herniou *et al.* 2013). The elements of *Microplitis demolitor* bracovirus (MdBV) within the *M. demolitor* genome have been described in depth, using the assembly named Mdem1 as a reference (Burke *et al.* 2014). Although the *M. demolitor* genome sequence was generated primarily to focus upon MdBV, there are few genomic resources available for braconid wasps and other parasitoids, making the wasp genome useful for researchers in other fields (*e.g.*, Geib *et al.* 2017; Bewick *et al.* 2017; Zhou *et al.* 2015). In this manuscript, we announce the full genome sequence of *M. demolitor* with an improved assembly and an annotated gene set for both wasp and viral genes. This publicly available genome assembly will continue to facilitate research on bracoviruses but also provide a resource to help address other questions specific to *M. demolitor* and to enable comparative analyses with other insect species.

## Materials and Methods

### Wasp samples

Wasp samples were derived from a culture maintained at the University of Georgia as described previously (Burke 2016). DNA was isolated from single and pooled male wasps with a high-salt precipitation method to maintain the integrity of high molecular weight DNA as described in Burke *et al.* (2014).

### Whole genome sequencing and assembly

In addition to the sequencing libraries reported in Burke *et al.* (2014) (180 bp, 1.5 kb, 5 kb, and 10 kb), a new 20 kb long-insert mate-pair library was constructed from pooled adult male DNA using Illumina’s Nextera Mate-Pairs Sample Prep Kit. All libraries were sequenced for 100 cycles on a HiSeq2000 using TruSeq chemistry. Raw reads were trimmed, filtered, and error-corrected as described in Burke *et al.* (2014). The Mdem1 assembly was further improved by additional scaffolding with the 20 kb Nextera mate-pair library and use of GapCloser v1.12 to close gaps generated in the scaffolding process with short paired read data (Luo *et al.* 2012). The genome assembly was screened by NCBI during the whole genome submission process to filter out adapter, vector, and other contaminant sequences. Methods used to generate RNASeq and viral DNA libraries and sequence data used for mapping have been described previously (Bitra *et al.* 2016; Burke and Strand 2014; Burke *et al.* 2014; Burke and Strand 2012; Burke 2016).

### Automated annotation of the M. demolitor genome

Structural and functional annotation of genes was performed with the NCBI Eukaryotic Genome Annotation Pipeline. This automated pipeline utilized short read transcript evidence from existing RNASeq data for *M. demolitor* (Burke *et al.* 2014), in addition to the MdBV proviral segments present in GenBank (Webb *et al.* 2006), NCBI RefSeq protein sets for *Fopius arisanus*, *Nasonia vitripennis* and *Apis mellifera* and 81,697 protein sequences from GenBank derived from the Insecta. Alignments were used to inform gene model prediction using the NCBI eukaryotic gene prediction tool *GNOMON*. Details of the annotation process can be accessed at: https://www.ncbi.nlm.nih.gov/genome/annotation_euk/process/. The completeness of the annotated gene set was analyzed by identifying the number of arthropod Benchmark Universal Single-Copy Orthologs (BUSCOs) (Simão *et al.* 2015). BUSCO v.1.1b1 was run on the RefSeq Gene set at the predicted peptide level (“-m OGS”). BUSCO results were compared to the RefSeq Gene sets for braconid species *F. arisanus* and *D. alloeum* as well as *Nasonia vitripennis*, for which a large portion of the genome is mapped to one of five chromosomes (Werren *et al.* 2010).

### Manual annotation of M. demolitor genes of viral origin

Manual verification or correction of nudivirus-like replication genes and proviral genes was performed using the *M. demolitor* jBrowse/Apollo instance hosted at the USDA National Agricultural Library i5k Workspace. Protein sequences from the previously published manually curated viral gene set from the Mdem1 assembly were aligned to the genome using a modified version of exonerate v. 2.3.0 in which the gff3 output is compatible with jBrowse for upload as a custom track (available at https://github.com/hotdogee/exonerate-gff3). Exonerate alignments were used as the basis for correction of existing gene models or addition of gene models missing in the Mdem2 annotation. The boundaries of proviral segments and replication units in the Mdem2 assembly were identified by searching for sequence motifs that define these regions, along with use of short read mapping data from existing deep sequencing data from MdBV viral DNA and DNA isolated from ovaries when replication and associated amplification of viral DNA is at its peak (Burke and Strand 2014; Burke *et al.* 2015). Short read data were filtered with the fastx toolkit to retain reads with a phred score equivalent >30 for >90% of bases within a read. Quality filtered reads from sequenced DNAs were mapped to the Mdem2 assembly using bwa mem v. 0.7.15. Similarly, quality-filtered RNASeq data from infected host cells of *Chrysodeixis includens* or *Trichoplusia ni* were mapped to the proviral regions of the Mdem2 assembly with hisat2 v.2.1.0 (Burke *et al.* 2014, Bitra *et al.* 2016, Kim *et al.* 2015). Any reads that did not map to the proviral segments were removed using samtools v.1.3.1 (Li *et al.* 2009).

### Data Availability

All raw sequencing data are available from the NCBI Sequence Read Archive (see Table 1 for accessions). The genome assembly, WGS Project AZMT02, is represented as BioSample SAMN02708865 with identical records in GenBank as accession GCA_000572035.2 and RefSeq as accession GCF_000572035.2 with the name Mdem2. All current annotations are associated with the RefSeq assembly as release 101. An FTP site for data download is at ftp://ftp.ncbi.nlm.nih.gov/genomes/Microplitis_demolitor/. NCBI’s Genome Data Viewer can be accessed at https://www.ncbi.nlm.nih.gov/genome/gdv/?acc=GCF_000806365.1&context=genome and an overview of release 100 annotations can be accessed at https://www.ncbi.nlm.nih.gov/genome/annotation_euk/Microplitis_demolitor/101. Curation of this assembly and consolidated sequence-based resources are hosted by the i5k Workspace (https://i5k.nal.usda.gov/) allowing visualization within jBrowse, manual curation with Apollo and other tools.

**Table 1:** Raw reads generated for assembly

SRA	Library type	Read pairs	Base pairs	Material
SRX610757	180 bp	138.9 M	17.5 Gb	Single adult male
SRX641381	1.5 kb	164.3 M	20.9 Gb	40 pooled adult male wasps
SRX641400	5 kb	53.6 M	7.1 Gb	40 pooled adult male wasps
SRX641403	10 kb	174.3 M	22.1 Gb	100 pooled adult male wasps
SRX641469	20 kb	230.2 M	28.3 Gb	100 pooled adult male wasps
SRX981480	5 kb	37.8 M	5.1 Gb	120 pooled adult male wasps

## Results And Discussion

In total, approximately 17.5 Gb of small-insert sequence data were generated from a single male adult wasp for the Mdem1 assembly, along with 129.4 Gb of data generated from larger insert libraries (1.5, 5, 10, and 20 kb insert sizes) for scaffolding purposes (Table 1). The 20 kb library derived data were not included in the previous assembly Mdem1. Assembly of these sequence data with SOAPdenovo resulted in a new assembly (Mdem2) that consisted of 1,794 scaffolds with an N50 size of 1.1 Mb and 27,508 contigs with an N50 of 14.12 kb (Table 2). The assembly was 241.2 Mb in total length, which has very good concordance with the genome size estimated by flow cytometry (241 +/− 6 Mb, Burke *et al.* 2014). Only 14.6% of the genome assembly was comprised of sequence gaps. The overall G + C nucleotide content was 33.1%. These assembly statistics are a large improvement over the Mdem1 assembly, with approximately 65% fewer scaffolds and an N50 size 3.6x longer (Table 2). Genome assemblies are available for three other braconid wasp species while sequences are available for a fourth (*Cotesia vestalis*) but have not been scaffolded. The Mdem2 assembly statistics are similar to these other braconids and *Nasonia vitripennis* (family Pteromalidae) in both genome size and G + C content (Table 2).

**Table 2:** Summary statistics for the Mdem2 assembly, the previous Mdem1 assembly, and select other parasitoid genomes

Species	Assembly	NCBI BioProject	Contig count (N50 kb)	Scaffold count (N50 Mb)	Total length (Mb)	GC (%)
*Microplitis demolitor*	Mdem2	PRJNA251518	27,508 (14.12)	1,794 (1.14)	241.2	33.1
*Microplitis demolitor*	Mdem1	PRJNA251518/PRJNA195937	36,718 (13.54)	5,174 (0.32)	250.5	33.1
*Fopius arisanus*	ASM80636v1	PRJNA258104	8,510 (51.90)	1,042 (0.98)	153.6	39.4
*Diachasma alloeum*	Dall1.0	PRJNA306876	25,534 (44.93)	3,968 (0.65)	388.8	39.1
*Cotesia vestalis*	ASM95615v1	PRJNA271135	9,156 (46.06)	—	186.1	30.6
*Nasonia vitripennis*	Nvit_2.1	PRJNA13660	25,484 (18.84)	6,169 (0.71)	295.8	40.6

Annotation using the NCBI Eukaryotic Annotation Pipeline yielded 12,755 genes or pseudogenes, including 12,144 containing protein coding regions. A total of 19,597 transcripts were annotated, with a mean of 1.54 (median 1) transcripts per gene (Table 3). Evidence for gene annotations were derived from RNA-Seq data from adult wasp ovaries, venom glands, and teratocytes, and larvae (Table 4) and proteins from related species, or *ab initio* evidence predicted by GNOMON. A large proportion of transcripts (16,219 of 18,586 (87.2%)) were fully supported with experimental evidence. A total of 526 non-coding genes were identified, including tRNAs, lncRNAs and others. Details of the annotation are presented in Table 3 as well as online at https://www.ncbi.nlm.nih.gov/genome/annotation_euk/Microplitis_demolitor/101/.

**Table 3:** Gene annotation summary statistics

Feature	Count	Mean length (bp)	Median length (bp)	Min length (bp)	Max length (bp)
Genes	12,670	10,107	3,317	71	539,413
All transcripts	19,597	2,356	1,840	71	44,309
mRNA	18,586	2,417	1,897	189	44,309
misc_RNA	224	3,134	2,043	106	19,088
tRNA	183	74	73	71	84
lncRNA	604	911	671	91	5,617
CDSs	18,586	1,916	1,401	105	43,512
Exons	78,299	365	211	2	14,552
Introns	64,033	1,911	183	30	353,735

**Table 4:** Raw reads from *M. demolitor* tissues or proviral segments used for annotation

Track name	Bioproject/ Sample ID	Nucleic acid	Number of reads	Percent aligned (quality-filtered) reads	Reference
Venom glands	PRJNA214515/SAMN02319525	RNA	136.4 M	95%	Burke and Strand 2014
Larvae	PRJNA214515/SAMN02319526	RNA	95.7 M	93%	Burke and Strand 2014
Teratocytes	PRJNA214515/SAMN02319527	RNA	99.0 M	88%	Burke and Strand 2014
Ovaries	PRJNA214515/SAMN02319528	RNA	103.3 M	90%	Burke and Strand 2012
Infected *C. includens* hemocytes	PRJNA285771/SAMN03758721	RNA	73.7 M	2%	Burke *et al.* 2014
Infected *C. includens*	PRJNA437008/SAMN08637637-	RNA	22.0 M	5%	Bitra *et al.* 2016
	SAMN08637639;				
	SAMN08637643-				
	SAMN08637645				
Infected *T. ni*	PRJNA437008/SAMN08637630, SAMN08637631, SAMN08637636,	RNA	37.5 M	4%	Bitra *et al.* 2016
	SAMN08637640-				
	SAMN08637642				
Bracovirus DNA	PRJNA319039/SAMN04875661	DNA	49.5 M	99%	Burke 2016; Burke *et al.* 2014
Ovary DNA	PRJNA319039/SAMN04875652	DNA	17.6 M	99%	Burke 2016; Burke *et al*. 2015

BUSCO analysis revealed that the *M. demolitor* genome assembly and annotation is very complete, with 97% of all BUSCOs conserved in Insecta identified in the protein-coding gene set (Table 5). Only 1.2% of BUSCOs were present as fragments in the *M. demolitor* annotation, and 0.7% were missing. These results are very similar to BUSCO analyses of other hymenopteran genomes (Table 5).

**Table 5:** BUSCO analysis of parasitoid wasp genomes

Species	CDS count	NCBI Refseq annotation release	Complete (% of total BUSCOs)	Fragmented (% of total BUSCOs)	Missing (% of total BUSCOs)
*M. demolitor*	18,586	101	2621 (97)	34 (1.2)	20 (0.7)
*F. arisanus*	18,906	100	2605 (97)	37 (1.3)	33 (1.2)
*D. alloeum*	19,692	100	2622 (98)	31 (1.1)	22 (0.8)
*N. vitripennis*	24,846	102	2585 (96)	34 (1.2)	50 (1.8)

As previously noted, BV genomes are integrated into the genomes of wasps. They also consist of two distinct components: proviral segments and nudivirus-like replication genes (Bézier *et al.* 2009; Burke *et al.* 2014, Figure 1). Expression of nudivirus-like replication genes in wasp ovaries results in formation of virions, while proviral segments, bounded by excision motifs targeted by specific nudivirus-like replication genes, are amplified in regions known as Replication Units (RUs), circularized and packaged into virions (Burke *et al.* 2013; Bézier *et al.* 2009; Burke *et al.* 2014; Annaheim and Lanzrein 2007; Savary *et al.* 1997; Bézier *et al.* 2013; Burke *et al.* 2015; Louis *et al.* 2013). This results in virions that package genes on proviral segments but lack all nudivirus-like replication genes. The genes located on proviral segments are often short and many contain introns (Webb and Strand 2005; Desjardins *et al.* 2008; Espagne *et al.* 2004). In contrast, no introns have been described for the nudivirus-like replication genes in bracoviruses (but see below) (Bézier *et al.* 2009; Burke *et al.* 2014).

**Figure 1:**
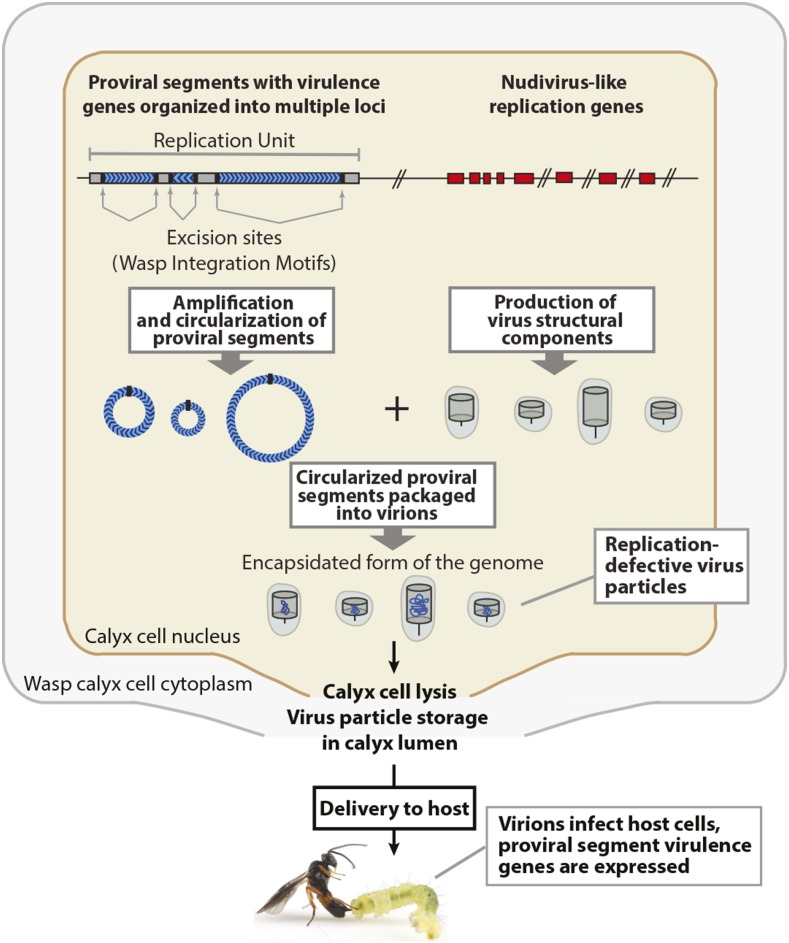
Schematic showing genome architecture and replication of bracoviruses. Proviral segments (shown as blue chevrons) reside in dispersed locations in the wasp genome and contain virulence genes. Proviral segments specifically amplify in the nuclei of calyx cells as Replication Units prior to segment excision and circularization. Nudivirus-like replication genes also reside in dispersed locations in the wasp genome (as indicated by hash marks) and encode proteins required to make virions. Expression of nudivirus-like replication genes in calyx cell nuclei results in formation of virions that package circularized proviral segments. Mature virions are released into the calyx region of wasp ovaries by lysis of calyx cells. Mature virions are replication-defective because the encapsidated proviral segments do not contain nudivirus-like replication genes. Wasps inject mature virions into host insects, which infect different cell types and express virulence genes that affect host immune defenses and growth.

*M. demolitor* genes of viral origin were previously described from manual annotation of the Mdem1 assembly (Burke *et al.* 2014). The genome contained 26 proviral segments that are amplified in eight replication units (Burke *et al.* 2015; Burke *et al.* 2014) located at 8 loci on *M. demolitor* scaffolds. 95 genes were identified in proviral segments, while 76 nudivirus-like replication genes were located on 30 different genome scaffolds. Evidence for these gene models was derived from RNASeq data from wasp cells and tissues as above and also MdBV infected hemocytes (Table 4, Burke *et al.* 2014). Only a single nudivirus-like replication gene was located on the same scaffold as a proviral segment (*HzNVorf93-like* and Segment T).

The Mdem2 automatic annotation performed by *GNOMON* correctly recovered 90% of the *M. demolitor* viral genes. Eighteen genes that were either missing or incorrectly annotated were manually corrected using alignment with older gene models in the *M. demolitor* Mdem2 jBrowse/Apollo instance hosted at the i5k Workspace. An additional four gene models (*lef-8*, *lef-9*, *HzNVorf128-like*, and *K425_12*) were edited to reflect the presence of introns that were previously unidentified.

The architecture of the proviral portion of the *M. demolitor* genome did not change appreciably between the Mdem1 and Mdem2 assemblies, with proviral segments still located in 8 loci across 9 scaffolds. Coordinates for proviral segments and replication units in the Mdem2 assembly are listed in Table 6. The nudivirus-like replication genes were located on 24 different scaffolds (5 fewer than in Mdem1 assembly). One major difference was that an additional link between proviral segments and nudivirus-like replication genes was identified. Locus 2, containing Segments V, W, E, C and X, was located on a 2.4 Mb scaffold approximately 75 kb away from the nudivirus-like gene *p74*, and more than 323 kb from several other nudivirus-like replication genes (*35a-8* to *35a-14*; *odv-e66-9* to *odv-e66-20*; *35a-6* and *35a-7*; and *helicase*). The entire set of proviral segments, replication units, and viral genes are available as gff3 and sequence files at AgDataCommons (http://dx.doi.org/10.15482/USDA.ADC/1432667) and can be uploaded as custom tracks for visualization at the i5k Workspace.

**Table 6:** Coordinates for boundaries of proviral segments and amplified replication units*^a^*

Segment	Locus	Orientation	Accession number	Segment start	Segment end	Amplification start	Amplification end
P	1	—	NW_014464280.1	204368	217086	202948	
K1	1	—	NW_014464280.1	217246	232492		
K	1	—	NW_014464280.1	>235072	245355		
Q	1	—	NW_014464280.1	245514	261072		
D	1	—	NW_014464280.1	261199	>274917		>274917
D	1	+	NW_014464327.1	325333	>327729	>327729	
B	1	+	NW_014464327.1	315630	321681		
A	1	+	NW_014464327.1	286830	312116		
L	1	+	NW_014464327.1	270462	285675		
F	1	—	NW_014464327.1	259195	>268430		
I	1	—	NW_014464327.1	245419	>258067*		
M	1	+	NW_014464327.1	228747	244908		
G	1	—	NW_014464327.1	215667	227866		214815
O	1	—	NW_014464327.1	>209595	214086	186757	214590
V	2	+	NW_014463818.1	>1914991*	1929656	1929927	
W	2	+	NW_014463818.1	1899259	>1901856*		
E	2	—	NW_014463818.1	1890266	1898510		
C	2	+	NW_014463818.1	1882990	1890217		
X	2	—	NW_014463818.1	1867002	1881299		1866484
N	3	—	NW_014463791.1	1514968	1532949	1511956	1533620
J	3	—	NW_014463791.1	1535308	1549002	1534400	1550957
H	4	+	NW_014463921.1	383509	394750	382579	395765
R	5	—	NW_014464373.1	36111	42534	32781	43423
S	6	—	NW_014463823.1	1794688	1806656	1780975	1810556
T	7	+	NW_014464188.1	421495	427196	421290	429489
U	8	+	NW_014463797.1	3414939	3421513	3414736	3423057

a Each proviral segment and its associated locus is listed in a row along with the *M. demolitor* genome scaffold where it is located. Scaffold accession numbers are indicated along with the coordinates for the boundaries of each proviral segment. Amplification start and end coordinates are listed for each RU that contains one segment. For multi-segment RUs, the amplification start and end coordinates correspond to the outermost segments. “>” signs indicate that gaps in scaffolds or scaffold termini prevent determination of segment or replication unit ends. “*” is similar to “>”, but segment ends are detected in smaller contigs that were not incorporated into scaffolds (*e.g.*, ends of Segments V and W are in NW_014463725.1, Mdem_contig_4120015, while the end of Segment I is in NW_014463324.1, Mdem_contig_4046930).

In addition to updating annotation of the regions of viral origin in the *M. demolitor* genome, we also consolidated all sequence-based resources we have available for *M. demolitor* on the jBrowse/Apollo instance of the genome hosted at the i5k Workspace. Genome resources include the most recent genome assembly (Mdem2) and gene sets from NCBI Annotation Release 101. Transcriptome data (in the form of BigWig coverage plots and mapped reads) are available for ovary, teratocyte, venom gland, and larval samples from wasps (Table 4). We have also contributed transcriptome data for all MdBV genes that are expressed in infected host caterpillars. These include the permissive host *Chrysodeixis includens* and the semipermissive host *Trichoplusia ni* data from hemocytes and whole body samples (Table 4). Finally, mapped DNA data are available from deep sequencing of DNAs isolated from MdBV virions and *M. demolitor* ovaries when proviral segment amplification is at its peak (Table 4). These data will facilitate the exploration of the evolution and function of MdBV and other viral symbionts in the future.

The *M. demolitor* genome described herein represents a high-quality assembly. The assembly of the genome has greatly benefitted from a sequencing strategy in which contigs were built from sequences derived from a single haploid male wasp, followed by scaffolding using sequence data from large-insert libraries. The Mdem1 assembly was also significantly improved with the addition of sequence data derived from a large insert (20kb) mate-pair library used in the Mdem2 assembly. The *M. demolitor* annotated gene set is similar to related genomes from select other parasitic Hymenoptera in terms of numbers of genes and estimated completeness.

The Mdem2 assembly also provides a more complete picture of the architecture of the MdBV genome in the wasp genome. While proviral segments share no similarity with sequences from pathogenic nudiviruses, prior results showing that the recognition of excision motifs on proviral segments by nudivirus-like integrases strongly suggests that the proviral segments and nudivirus-like replication genes have shared ancestry (Burke *et al.* 2013). While it is unclear how genome rearrangement of the viral genome was achieved in the wasp genome, the physical location of several nudivirus-like replication genes and proviral segments in neighboring regions of *M. demolitor* chromosomes provides further evidence for their shared evolutionary history (Strand and Burke 2015). Future assemblies with new long-read sequencing technologies generating chromosome-length scaffolds will provide further insight into the location of viral genome components relative to each other. These data will help to determine whether proviral segment loci and nudivirus-like replication genes are limited to either single or multiple chromosomes in the wasp genome, which will provide information about the events leading to the inception of viral sequences in the wasp genome and their maintenance over time.
